# Basal cell carcinoma: PD-L1/PD-1 checkpoint expression and tumor regression after PD-1 blockade

**DOI:** 10.1186/s40425-017-0228-3

**Published:** 2017-03-21

**Authors:** Evan J. Lipson, Mohammed T. Lilo, Aleksandra Ogurtsova, Jessica Esandrio, Haiying Xu, Patricia Brothers, Megan Schollenberger, William H. Sharfman, Janis M. Taube

**Affiliations:** 1 Department of Oncology, Johns Hopkins University School of Medicine, Sidney Kimmel Comprehensive Cancer Center, and Bloomberg ~ Kimmel Institute for Cancer Immunotherapy, Baltimore, MD USA; 2 Department of Pathology, Johns Hopkins University School of Medicine, Sidney Kimmel Comprehensive Cancer Center, and Bloomberg ~ Kimmel Institute for Cancer Immunotherapy, Baltimore, MD USA; 3 Department of Dermatology, Johns Hopkins University School of Medicine, Sidney Kimmel Comprehensive Cancer Center, and Bloomberg ~ Kimmel Institute for Cancer Immunotherapy, Baltimore, MD USA; 4 Melanoma and Cancer Immunology Programs, Johns Hopkins University School of Medicine, 1550 Orleans Street, Room 507, Baltimore, MD 21231 USA

**Keywords:** PD-L1, Anti-PD-1, Basal cell carcinoma, Hedgehog, Pembrolizumab

## Abstract

Monoclonal antibodies that block immune regulatory proteins such as programmed death-1 (PD-1) have demonstrated remarkable efficacy in controlling the growth of multiple tumor types. Unresectable or metastatic basal cell carcinoma, however, has largely gone untested. Because PD-Ligand-1 (PD-L1) expression in other tumor types has been associated with response to anti-PD-1, we investigated the expression of PD-L1 and its association with PD-1 expression in the basal cell carcinoma tumor microenvironment. Among 40 basal cell carcinoma specimens, 9/40 (22%) demonstrated PD-L1 expression on tumor cells, and 33/40 (82%) demonstrated PD-L1 expression on tumor-infiltrating lymphocytes and associated macrophages. PD-L1 was observed in close geographic association to PD-1+ tumor infiltrating lymphocytes. Additionally, we present, here, the first report of an objective anti-tumor response to pembrolizumab (anti-PD-1) in a patient with metastatic PD-L1 (+) basal cell carcinoma, whose disease had previously progressed through hedgehog pathway-directed therapy. The patient remains in a partial response 14 months after initiation of therapy. Taken together, our findings provide a rationale for testing anti-PD-1 therapy in patients with advanced basal cell carcinoma, either as initial treatment or after acquired resistance to hedgehog pathway inhibition.

## Background

Basal cell carcinoma (BCC) is the most common human cancer, though most tumors are eradicated using locally-directed therapies [1]. In the small minority of patients that develop surgically unresectable locally-advanced or metastatic disease, currently available systemic therapies, such as agents that curtail aberrant signaling along the hedgehog (Hh) pathway, are frequently ineffective in bringing about durable anti-tumor responses [2–6].

Over the past several years, monoclonal antibodies that block immune checkpoint proteins (e.g., anti-programmed death-1 (PD-1), PD-Ligand-1 (PD-L1)), have demonstrated remarkable efficacy in controlling the growth of multiple tumor types [7]. In several studies, the expression of PD-L1 – a major ligand of PD-1 – in the tumor microenvironment has been associated with an increased likelihood of an anti-tumor response to anti-PD-1. BCC, however, has largely gone untested and little is known about the expression of immunoregulatory molecules in the BCC microenvironment or about the capacity of PD-1-pathway-directed therapies to trigger an effective anti-tumor response [8–10].

In the current study, we characterized PD-L1 and PD-1 expression patterns in the tumor microenvironment of 40 archived BCC specimens. Additionally, we studied a pre-treatment tumor specimen and clinical and radiographic response characteristics from one patient who experienced a durable, objective anti-tumor response to pembrolizumab (anti-PD-1) monotherapy.

## Materials and methods

Following Institutional Review Board approval, 40 surgical pathology specimens from 40 unique patients with BCC were identified from the Johns Hopkins Hospital surgical pathology archives. Slides were reviewed by a board-certified dermatopathologist (JMT) to confirm the diagnosis, and one representative paraffin block was chosen for PD-L1 and PD-1 immunohistochemistry (IHC). PD-L1 expression was assessed using the murine anti-human PD-L1 monoclonal antibody 5H1 (from Lieping Chen, Yale University, New Haven, CT) at a concentration of 0.1 μg/mL, as previously described [11]. PD-L1 expression on tumor cells and immune cells were scored separately. Cases demonstrating at least 5% membranous (cell surface) expression of PD-L1 were considered positive. Immunohistochemistry for PD-1 was performed using a primary mouse anti-human mAb (clone Nat105, at 1:1000), following an antigen retrieval of 10 min in citrate buffer, pH 6.0 at 120 C. A secondary anti-mouse IgG1 antibody was used at 1.0 μg/ml. Amplification was performed by using Perkin Elmer biotin TSA, Dako streptavidin HRP and then visualized by DAB staining. The geographic association between PD-L1 and PD-1 expression in the tumor microenvironment was also assessed. For the specimen from the patient demonstrating a response to anti-PD-1, an extended panel of IHC markers (CD4, CD8, TIA-1 and CD68) was performed according to standard automated methods.

## Results

**Fig. 1:**
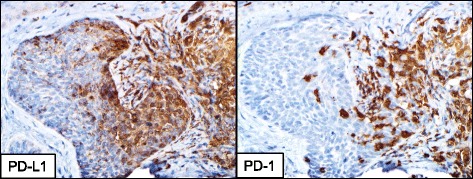
Geographic co-localization of PD-L1+ tumor cells and associated immune cells with PD-1+ infiltrating immune cells in BCC. PD-1, programmed death-1; PD-L1, programmed death ligand-1. 200× original magnification, all panels

Forty specimens from 40 unique patients were evaluable. All were primary lesions that were locally aggressive or recurrent. The tumors ranged in size from 2 to15 cm in greatest dimension. Nine of forty (22%) demonstrated PD-L1 expression on tumor cells, and 33/40 (82%) demonstrated PD-L1 expression on tumor-infiltrating lymphocytes (TIL) and associated macrophages. All (40/40, 100%) of cases had varying degrees of either true TIL present or lymphocytes present in the immediate extratumoral stroma. PD-1 expression was seen on lymphocytes in the tumor microenvironment of each case, including the 16/40 (40%) cases that had only rare lymphocytes present. All (33/33) cases with PD-L1 expression on tumor or macrophages showed an association with PD-1+ TIL (Fig. 1). There were no cases that demonstrated constitutive PD-L1 expression, independent of TIL [11].

## Case presentation

A 67-year-old woman presented in 2007 with a large BCC of the skin of the right posterior neck. She underwent wide local excision followed by adjuvant electron radiotherapy. In 2011 she developed a surgically unresectable recurrence and began therapy on a phase 1 clinical trial of the Hh pathway inhibitor saridegib (IPI-926; clinicaltrials.gov ID# NCT00761696). Although radiographically her best overall response per RECIST criteria was stable disease, clinically, her symptoms related to tumor growth improved dramatically for several months, then worsened about 12 months after having initiated therapy.

She then pursued treatment on a phase 1 clinical trial of AZD6244 (MEK inhibitor) and IMC-A12 (a fully human IgG1 monoclonal antibody directed against insulin-like growth factor-1 receptor (IGF-1R); clinicaltrials.gov ID# NCT01061749). Her disease progressed within 6 months, requiring palliative radiotherapy.

In late 2014 her neck pain increased and CT scans demonstrated multiple lung metastases. Re-initiation of Hh inhibition was considered but, based on previous clinical experience, the likelihood of anti-tumor activity was felt to be low [12]. The patient preferred to avoid the toxicity associated with standard cytotoxic chemotherapy.

Although we had not yet assessed PD-L1 expression in this patient’s tumor, support for the use of PD-1 pathway-directed therapy came from pre-clinical studies demonstrating that BCC typically caries a high genetic mutation burden, a characteristic associated with response to PD-1 blockade in other tumor types [13, 14]. Additionally, BCCs undergoing spontaneous regression (presumably immune-mediated) contain elevated levels of T-helper type-1 (Th1) cytokines (e.g., interferon gamma) and infiltrating activated T cells [15, 16].

**Fig. 2:**
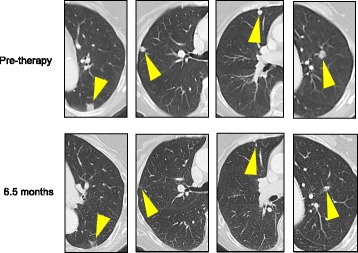
Partial response of metastatic basal cell carcinoma to pembrolizumab (anti-PD-1). Computed tomography (CT) scans performed pre-therapy (*top row*) and 6.5 months after initiating pembrolizumab (anti-PD-1, *bottom row*) demonstrate regression of basal cell carcinoma lung metastases. *Yellow arrowheads* indicate sites of metastases

Pembrolizumab (anti-PD-1) was administered at 2 mg/kg IV every 3 weeks beginning in December 2015. The following month, the patient reported that her pain medication requirement had decreased substantially. CT scans performed 4 months into therapy demonstrated a partial response (PR) to therapy per RECIST 1.1 criteria (Fig. 2). Fourteen months into therapy, a PR is ongoing. The patient has experienced subclinical hypothyroidism, possibly drug-related.

**Fig. 3:**
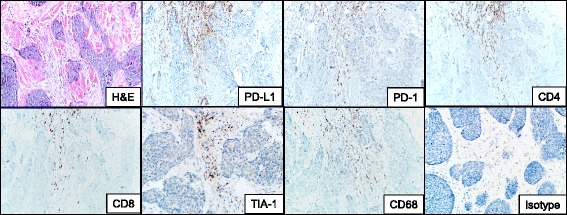
Immune elements in the microenvironment of a pre-treatment basal cell carcinoma from a patient who responded to anti-PD-1 therapy. The immune infiltrate abuts the tumor islands and is composed of a mixture of CD4 and CD8+ T-cells at a ratio of approximately 2:1. The CD8 cells are cytotoxic, as supported by the punctate cytoplasmic TIA-1 immunostaining. The lymphocytic infiltrate is accompanied by CD68+ macrophages. PD-1 is seen on approximately half of the lymphocytes present, and is immediately adjacent to PD-L1 expression in the tumor microenvironment, consistent with an immune microenvironment primed for potential response to PD-1/PD-L1 checkpoint blockade. PD-L1 is expressed predominantly on immune cells, rather than tumor cells in this example. H & E, hematoxylin and eosin, PD-(L)1, programmed death-(Ligand)1. 200× original magnification, all panels

Immunohistochemical evaluation of the patient’s pre-treatment BCC demonstrated PD-L1 expression on immune cells but not tumor cells (Fig. 3). This immune infiltrate was composed of a mixture of CD4 and CD8+ T cells as well as CD68+ macrophages. Approximately 50% of the lymphocytes present expressed PD-1. Immunohistochemical stains for HLA-I and II were not performed, as they are not yet fully validated in our laboratories.

## Discussion

The biology of BCC presents opportunities for both immune-mediated tumor regression and escape from immune surveillance. Factors that may increase BCC’s immunogenicity include its high rate of cancer-testis antigen expression [17], copious numbers of tumor-infiltrating CD8+ T cells [18], and a genetic mutational burden which is among the highest reported in any human cancer type [14, 19–21]. Indeed, many of the cancers against which immune checkpoint therapy is effective (e.g., melanoma, non-virus-associated Merkel cell carcinoma and microsatellite unstable neoplasms) harbor large genetic mutational loads [22, 23]. A recent case report from Ikeda and colleagues describes a near-complete response to nivolumab (anti-PD-1) in a patient with metastatic BCC whose tumor carried a particularly high mutational burden (450 mutations per megabase) [9]. Conversely, BCCs demonstrate low levels of MHC-I expression [24] and contain abundant regulatory T cells in the tumor microenvironment [18], both of which may suppress anti-tumor immunity and allow for immune escape.

The administration of various anti-neoplastic therapies may alter the immunological profile in BCCs. For example, application of imiquimod to BCC results in upregulation of MHC-I expression [17]. Likewise, administration of the Hh pathway inhibitors vismodegib or sonidegib to patients with BCC triggers increases in tumor-infiltrating T cells and tumor cell MHC-I expression [25]. Despite these seemingly beneficial immunological effects, Hh pathway inhibitors have demonstrated sub-optimal objective response rates of 15–60%, with median durations of response often <12 months [3, 4, 6, 26].

Taken together, these findings suggest that appropriately-activated immune responses directed against BCC may control tumor growth. In the current study, we present a pre-clinical rationale for, and clinical evidence of, potential long-term anti-tumor immunity after administration of anti-PD-1, an immune checkpoint blocker. Agents targeting immune checkpoints represent “common denominator” therapies which can bring about durable anti-tumor responses in patients with multiple tumor types [27]. PD-L1 expression on tumor cells and immune cells has been shown to enrich for response to anti-PD-1/L1 in various solid malignancies [7]. Here, we demonstrate prominent expression of two of the checkpoint pathway’s component molecules, PD-1 and PD-L1, in BCC. Moreover, the cases in the current series exhibit PD-1 and PD-L1 expression in close geographic proximity to each other, a pattern consistent with adaptive (i.e., interferon-gamma-mediated) immune resistance [11]. The adaptive pattern of PD-L1 expression in the tumor microenvironment can be further accentuated by amplification of the 9p24.1 locus, which contains PD-L1, −L2 and JAK2, the latter of which is an interferon-gamma-responsive element. This alteration was first described in Hodgkin lymphoma [28], then recently illustrated in a case report of a patient with BCC whose tumors regressed after administration of anti-PD-1 [9]. Importantly, mutational burden and PD-1/PD-L1 expression in the tumor microenvironment do not appear to be directly linked [29]. Thus, the current report highlights a novel biomarker suggesting potential efficacy of anti-PD-1 in patients with advanced BCC.

Of note, the immune marker panel used in our study was intended to support the aim of our investigation: to explore expression of molecules comprising the PD-L1 axis in BCC as a rationale for clinical application of anti-PD-1/PD-L1 agents. Development of an optimized multiplex biomarker panel for BCC will likely include assessment of additional immunoregulatory elements (e.g., HLA-I and -II expression status), and will require a large cohort of patients.

In conclusion, our laboratory and clinical findings suggest that PD-1 blockade should be considered as salvage therapy in patients with advanced BCC whose disease has progressed after standard Hh pathway inhibition. Our results also provide a rationale for ongoing clinical trials of PD-1 pathway blockade agents in patients with advanced BCC, either as first-line therapy or in combination with or after treatment with Hh inhibitors (e.g., NCT02690948).

## Abbreviations

BCC: Basal cell carcinoma
Hh: Hedgehog
PD-1: Programmed Death-1
PD-L1: Programmed Death Ligand-1
